# Remdesivir-Induced Marked Sinus Bradycardia in COVID-19

**DOI:** 10.7759/cureus.27249

**Published:** 2022-07-25

**Authors:** Taimoor Ahmed, Samra Haroon Lodhi, Taha Ahmed

**Affiliations:** 1 Internal Medicine, Mayo Hospital, Lahore, PAK; 2 Internal Medicine, University of Kentucky College of Medicine, Lexington, USA

**Keywords:** telemetry, electrocardiogram (ecg/ekg), bradycardia, covid 19, remdesivir

## Abstract

Remdesivir has been extensively employed during the coronavirus disease 2019 (COVID-19) pandemic as it has proven to be efficacious against the causative SARS-CoV-2. However, there is not much evidence on the cardiovascular adverse effect profile of remdesivir. In addition, limited data support the occurrence of sinus bradycardia associated with remdesivir. Herein we chronicle a clinical encounter of a patient suffering from COVID-19 whose clinical course was complicated by marked sinus bradycardia that began acutely after remdesivir initiation and resolved on cessation of the medication. The patient denied symptoms and completed a 5-day course with a resolution of bradycardia on completion of medication. We suggest that the physicians be cognizant of this rare side effect of remdesivir and suggest a continuation of this medication unless symptomatic bradycardia precludes management.

## Introduction

On October 22, 2020, remdesivir was the first antiviral drug approved by the US FDA to treat coronavirus disease 2019 (COVID-19) in patients requiring hospitalization [1]. It has been used compassionately in the US since the first confirmed case of COVID-19, leading to a marked improvement in patients' clinical status within 24-48 hours [2]. However, little is known about the cardiovascular safety profile of the medication, and a few reports showing the association of remdesivir with sinus bradycardia have emerged recently [3, 4]. We present a case of a female patient who presented with worsening COVID-19 and developed asymptomatic sinus bradycardia soon after IV remdesivir was initiated. The patient improved clinically and bradycardia resolved after completing a 5-day course of remdesivir.

## Case presentation

**Figure 1:**
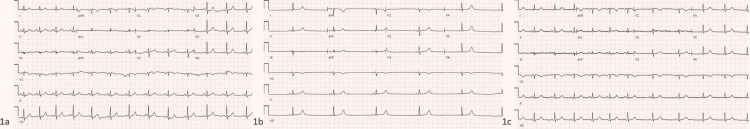
12-Lead electrocardiogram. (a) Normal sinus rhythm with a heart rate of 75 beats per minute on presentation (bpm); (b) sinus bradycardia with a heart rate of 33 bpm on day 3 of remdesivir therapy; (c) normal sinus rhythm with a heart rate of 62 bpm on completion of remdesivir therapy.

A 60-year-old female presented to the ED with complaints of fever, weakness, and anorexia secondary to COVID-19 infection. The patient did not have a significant past medical history. The patient reported fatigue for 10 days after a recent air trip. She went back to working as a clerk, but her condition worsened with the onset of nonproductive cough, progressive weakness, 103 C fever, severe headaches, shortness of breath, and watery diarrhea. She was diagnosed with COVID-19 at her local urgent care center. She had been using acetaminophen and over-the-counter nasal sprays with no relief, and three days later, she presented to the hospital with worsening of her symptoms. On presentation, the vital signs showed a temperature of 100.4 C, heart rate of 75 beats per minute, blood pressure of 92/51 mmHg, and respiratory rate of 20 per minute with oxygen saturation of 88% while resting on room air. Complete blood count, complete metabolic panel, and cardiac enzymes were unremarkable. Significant laboratory findings included an elevated C-reactive protein of 116 mg/L (normal range <9 mg/L), lactate dehydrogenase of 368 U/L (normal range: 116-250 U/L), and D-dimer of 1.42 ug/mL (normal range <0.51 ug/mL). An ECG showed a normal sinus rhythm (Figure 1a). A chest X-ray was significant for mild interstitial opacification and ground-glass opacities in the left upper lobe.

**Figure 2:**
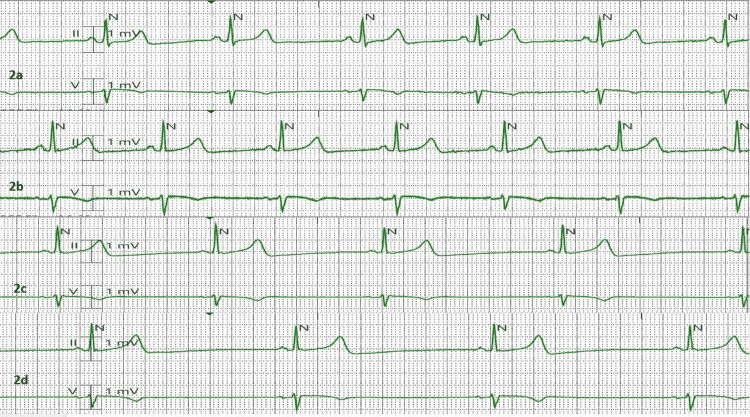
Telemetry strips on (a) Day 1, (b) Day 2, (c) Day 3, and (d) Day 4 of remdesivir therapy with progressive marked sinus bradycardia.

IV fluids and acetaminophen were administered for hypotension and fever, respectively. As the patient was persistently hypoxic, requiring supplemental oxygen via nasal cannula to maintain oxygen saturations greater than 90% and tachypnea, she was commenced on IV dexamethasone and remdesivir. Over the next two days, the patient's heart rate varied between 40 and 60 beats per minute (Figure 2a-2b).

On day 3 of hospitalization, the rapid response team was called for bradycardia with a heart rate of 30 beats per minute on a telemetry monitor (Figure 2c). The patient was completely asymptomatic, and a 12-lead electrocardiogram showed marked sinus bradycardia (Figure 1b). The rapid response was called off, and the patient was kept on telemetry monitoring (Figure 2d). Brain natriuretic peptide was normal and thyroid-stimulating hormone level (TSH) was low at 0.15 uIU/mL (normal range 0.4-4.2 uIU/mL) with a normal free thyroxine (T4) level of 1.5 ng/dL (normal 0.8-1.7 ng/mL). She responded well to the medical therapy with clinical improvement, and her inflammatory markers trended downwards. As she finished a 5-day course of remdesivir, her heart rate went back up between 60 and 70 beats per minute (Figure 1c). Dexamethasone was switched to oral, and she was discharged on day 5 of hospitalization with instructions to quarantine at home. At one month follow-up, the patient reported substantially improved shortness of breath and fatigue. In addition, an ECG showed normal sinus rhythm with a heart rate of 62 beats per minute and a normalized TSH of 0.90 uIU/mL, and free T4 of 1.2 ng/mL.

## Discussion

Bradycardia is traditionally defined as a heart rate of fewer than 60 beats per minute [5]. Common causes of sinus bradycardia include the use of atrioventricular blocking medications (beta-blockers, calcium channel blockers, amiodarone, digoxin), increased vagal tone (in athletes, during sleep, inferior myocardial infarction), metabolic causes (hypoxia, sepsis, myxedema, hypothermia, low glucose), obstructive sleep apnea (OSA), and increased intracranial pressure. Our patient was not on any medications at home and had a sedentary lifestyle, with relatively normal thyroid studies on follow-up, high body temperatures, and normal glucose during the hospitalization. Nevertheless, the bradycardia persisted while she was awake and was clinically improving. Furthermore, her screening score for OSA was low. Therefore, after ruling out all potential causes and due to the temporal relationship, we presume remdesivir to be the culprit. Remdesivir is a broad-spectrum antiviral agent with activity against RNA viruses. It is an adenosine nucleotide prodrug and, upon cellular uptake, is metabolized to a nucleoside monophosphate (NM) intermediate. The NM is phosphorylated by cellular kinases to the pharmacologically active nucleoside triphosphate metabolite. Remdesivir triphosphate (RDV-TP) acts as an analog of adenosine triphosphate (ATP) and competes with ATP for RNA incorporation by SARS-CoV2 RNA-dependent RNA polymerase, resulting in delayed viral RNA replication. RDV-TP also acts as a substrate for several viral RNA-dependent RNA polymerases, which it inhibits. However, RDV-TP is also a weak inhibitor of mammalian DNA and RNA polymerases, including human mitochondrial RNA polymerase. Inactive ingredients in the remdesivir injection include sulfobutylether-ß-cyclodextrin sodium salt (SBECD), water, hydrochloric acid, and/or sodium hydroxide for pH adjustment [6].

Despite the compassionate use of remdesivir for hospitalized patients with COVID-19, there is a scarcity of data on the drug's cardiac side effect profile. A randomized controlled trial (RCT) during the Ebola pandemic of 2016 showed that one out of 673 patients had bradycardia and hypotension [7]. During the COVID-19 pandemic, RCTs showed that six out of 53 patients in one trial developed atrial fibrillation, and one out of 233 patients developed cardiac arrest in another trial on remdesivir therapy. Recent reports have documented the occurrence of symptomatic sinus bradycardia resulting in cessation of the medication [8, 9]. Possible mechanisms include inhibition of human mitochondrial RNA polymerase, leading to mitochondrial dysfunction, resemblance to adenosine triphosphate, and affinity for A1 receptors causing changes in atrioventricular nodal conduction [4]. However, asymptomatic sinus bradycardia has not been associated with adverse outcomes [10]. Furthermore, one single-center study demonstrated a more favorable disease course in patients experiencing bradycardia during remdesivir treatment [11]. Another single-center study showed no significant impact of bradycardia with remdesivir treatment on ICU stay and in-hospital mortality [12]. Our patient was continued on a 5-day remdesivir course given the clinical benefit in this patient cohort. With the recent approval of remdesivir by the US FDA and due to the rising number of COVID-19 cases in the US, we presume that the use of remdesivir is on the rise. We aim to make the healthcare workers wary of this rare side effect associated with remdesivir. We recommend dynamic surveillance with clinical and telemetry monitoring for patients on remdesivir therapy and advise continuation of the medication unless symptomatic bradycardia precludes treatment.

## Conclusions

Remdesivir is the first antiviral agent to be approved by the US FDA for the treatment of COVID-19, as it has shown clinical efficacy in phase III clinical trials. However, there is a paucity of data on the cardiovascular safety profile of the drug. We present a case of marked sinus bradycardia in a COVID-19 patient with no risk factors for bradycardia, which resolved on completion of therapy. To our knowledge, the patient did not receive any other medication that could have caused the bradycardia. While bradycardia with remdesivir treatment has been reported with no significant difference in short-term outcomes, supporting the safe use of the medication, larger-scale studies, particularly in patients with pre-existing cardiac conduction disorders, are instructive. 
